# 5-Bromo-2,7-dimethyl-3-methyl­sulfinyl-1-benzofuran

**DOI:** 10.1107/S1600536809034011

**Published:** 2009-08-29

**Authors:** Pil Ja Seo, Hong Dae Choi, Byeng Wha Son, Uk Lee

**Affiliations:** a Department of Chemistry, Dongeui University, San 24 Kaya-dong Busanjin-gu, Busan 614-714, Republic of Korea; b Department of Chemistry, Pukyong National University, 599-1 Daeyeon 3-dong, Nam-gu, Busan 608-737, Republic of Korea

## Abstract

In the title compound, C_11_H_11_BrO_2_S, the O atom and the methyl group of the methyl­sulfinyl substituent are located on opposite sides of the plane of the benzofuran fragment. The crystal structure is stabilized by non-classical inter­molecular C—H⋯O hydrogen bonding, and by inter­molecular C—Br⋯π inter­actions, with C—Br⋯*Cg* = 3.629 Å (*Cg* is the centroid of the benzene ring).  In addition, the crystal structure exhibits aromatic π–π interactions between the furan rings of neighbouring molecules [centroid–centroid distance = 4.206 (6) Å].

## Related literature

For the crystal structures of similar 5-halo-2-methyl-3-methyl­sulfinyl-1-benzofuran derivatives. see: Choi *et al.* (2007**a* ▶,b* ▶). For natural products with a benzofuran ring, see: Akgul & Anil (2003 ▶); von Reuss & König (2004 ▶). For the pharmacological activity of benzofuran compounds, see: Howlett *et al.* (1999 ▶).
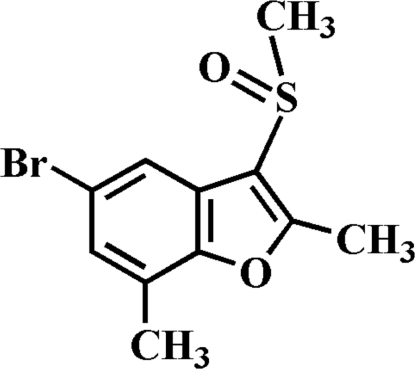


## Experimental

### 

#### Crystal data

C_11_H_11_BrO_2_S
*M* *_r_* = 287.17
Monoclinic, 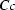

*a* = 16.929 (2) Å
*b* = 5.1001 (6) Å
*c* = 13.800 (2) Å
β = 106.962 (2)°
*V* = 1139.7 (3) Å^3^
*Z* = 4
Mo *K*α radiation
μ = 3.77 mm^−1^
*T* = 173 K
0.60 × 0.30 × 0.15 mm

#### Data collection

Bruker SMART CCD diffractometer
Absorption correction: multi-scan (*SADABS*; Sheldrick, 2000 ▶) *T*_min_ = 0.211, *T*_max_ = 0.602
3270 measured reflections
1832 independent reflections
1760 reflections with *I* > 2σ(*I*)
*R*_int_ = 0.111

#### Refinement

*R*[*F* ^2^ > 2σ(*F* ^2^)] = 0.040
*wR*(*F* ^2^) = 0.102
*S* = 1.06
1832 reflections
138 parameters
2 restraints
H-atom parameters constrained
Δρ_max_ = 1.31 e Å^−3^
Δρ_min_ = −0.82 e Å^−3^
Absolute structure: Flack (1983 ▶), 582 Friedel pairs
Flack parameter: −0.003 (12)

### 

Data collection: *SMART* (Bruker, 2001 ▶); cell refinement: *SAINT* (Bruker, 2001 ▶); data reduction: *SAINT*; program(s) used to solve structure: *SHELXS97* (Sheldrick, 2008 ▶); program(s) used to refine structure: *SHELXL97* (Sheldrick, 2008 ▶); molecular graphics: *ORTEP-3* (Farrugia, 1997 ▶) and *DIAMOND* (Brandenburg, 1998 ▶); software used to prepare material for publication: *SHELXL97*.

## Supplementary Material

Crystal structure: contains datablocks global, I. DOI: 10.1107/S1600536809034011/hg2560sup1.cif

Structure factors: contains datablocks I. DOI: 10.1107/S1600536809034011/hg2560Isup2.hkl

Additional supplementary materials:  crystallographic information; 3D view; checkCIF report

## Figures and Tables

**Table 1:** Hydrogen-bond geometry (Å, °)

*D*—H⋯*A*	*D*—H	H⋯*A*	*D*⋯*A*	*D*—H⋯*A*
C11—H11*B*⋯O2^i^	0.96	2.42	3.242 (7)	143

Symmetry code: (i) 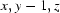.

**Fig. 1.**
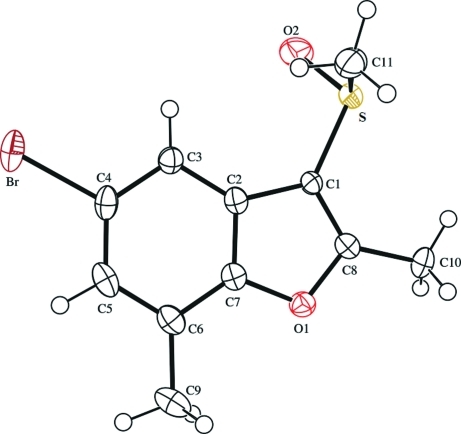
The molecular structure of the title compound with the atom numbering scheme. Displacement ellipsoids are drawn at the 50% probability level. H atoms are presented as a small cycles of arbitrary radius.

**Fig. 2.**
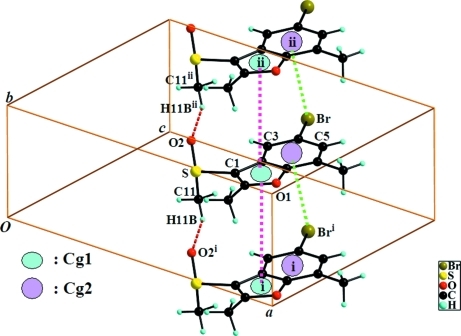
C–H···O, C–Br···π and π–π interactions (dotted lines) in the title compound. Cg denotes the ring centroids. [Symmetry codes: (i) x, - 1 + y, z; (ii) x, 1 + y, z.]

## supplementary crystallographic information

### Comment

Benzofuran ring systems are widely occurring in natural products (Akgul & Anil, 2003; von Reuss & König, 2004) and in synthetic substances which exhibit a variety of pharmacological properties (Howlett *et al.*, 1999). As a part of our continuing studies of the effect of side chain substituents on the solid state structures of 5-halo-2-methyl-3-methylsulfinyl-1-benzofuran analogues (Choi *et al.*, 2007*a,b*), the crystal structure of the title compound has been determined (Fig. 1).

The benzofuran unit is essentially planar, with a mean deviation of 0.009 (4) Å from the least-squares plane defined by the nine constituent atoms. The molecular packing (Fig. 2) is stabilized by non-classical intermolecular C–H···O hydrogen bond between the methyl H atom of the methylsulfinyl substituent and the oxygen of the S═O unit, with a C11–H11B···O2^i^ (Table 1). The crystal packing (Fig. 2) is further stabilized by intermolecular C–Br···π interaction between the Br atom and the benzene ring of an adjacent molecule, with a C4–Br···*Cg*2^ii^ distance of 3.629Å (*Cg*2 is the centroid of the C2–C7 benzene ring). Additionally, the molecular packing (Fig. 2) exhibits aromatic π–π interaction between the furan rings of neighbouring molecules, with a Cg1···Cg1^i^ distance of 4.206 Å (Cg1 is the centroid of the C1/C2/C7/O1/C8 furan ring).

### Experimental

77% 3-chloroperoxybenzoic acid (247 mg, 1.1 mmol) was added in small portions to a stirred solution of 5-bromo-2,7-dimethyl-3-methylsulfanyl-1-benzofuran (287 mg, 1.0 mmol) in dichloromethane (30 ml) at 273 K. After being stirred for 3 h at room temperature, the mixture was washed with saturated sodium bicarbonate solution and the organic layer was separated, dried over magnesium sulfate, filtered and concentrated in vacuum. The residue was purified by column chromatography (ethyl acetate) to afford the title compound as a colorless solid [yield 83%, m.p. 411-412 K; *R*_f_ = 0.33 (ethyl acetate)]. Single crystals suitable for X-ray diffraction were prepared by evaporation of a solution of the title compound in chloroform at room temperature.

### Refinement

All H atoms were geometrically positioned and refined using a riding model, with C–H = 0.93 Å for the aryl and 0.96 Å for the methyl H atoms. *U*_iso_(H) = 1.2Ueq(C) for the aryl and 1.5*U*_eq_(C) for the methyl H atoms.

### Crystal data

**Table:**

C_11_H_11_BrO_2_S	*F*(000) = 576
*M**_r_* = 287.17	*D*_x_ = 1.674 Mg m^−^^3^
Monoclinic, *C**c*	Mo *K*α radiation, λ = 0.71073 Å
Hall symbol: C -2yc	Cell parameters from 2606 reflections
*a* = 16.929 (2) Å	θ = 2.5–27.4°
*b* = 5.1001 (6) Å	µ = 3.77 mm^−^^1^
*c* = 13.800 (2) Å	*T* = 173 K
β = 106.962 (2)°	Block, colorless
*V* = 1139.7 (3) Å^3^	0.60 × 0.30 × 0.15 mm
*Z* = 4	

### Data collection

**Table:**

Bruker SMART CCD diffractometer	1832 independent reflections
Radiation source: fine-focus sealed tube	1760 reflections with *I* > 2σ(*I*)
graphite	*R*_int_ = 0.111
Detector resolution: 10.0 pixels mm^-1^	θ_max_ = 27.0°, θ_min_ = 2.5°
φ and ω scans	*h* = −21→19
Absorption correction: multi-scan (*SADABS*; Sheldrick, 2000)	*k* = −6→6
*T*_min_ = 0.211, *T*_max_ = 0.602	*l* = −13→17
3270 measured reflections	

### Refinement

**Table:**

Refinement on *F*^2^	Secondary atom site location: difference Fourier map
Least-squares matrix: full	Hydrogen site location: difference Fourier map
*R*[*F*^2^ > 2σ(*F*^2^)] = 0.040	H-atom parameters constrained
*wR*(*F*^2^) = 0.102	*w* = 1/[σ^2^(*F*_o_^2^) + (0.071*P*)^2^ + 0.1*P*] where *P* = (*F*_o_^2^ + 2*F*_c_^2^)/3
*S* = 1.06	(Δ/σ)_max_ = 0.001
1832 reflections	Δρ_max_ = 1.31 e Å^−^^3^
138 parameters	Δρ_min_ = −0.82 e Å^−^^3^
2 restraints	Absolute structure: Flack (1983), 582 Friedel pairs
Primary atom site location: structure-invariant direct methods	Flack parameter: −0.003 (12)

### Special details

**Table:**

Geometry. All esds (except the esd in the dihedral angle between two l.s. planes) are estimated using the full covariance matrix. The cell esds are taken into account individually in the estimation of esds in distances, angles and torsion angles; correlations between esds in cell parameters are only used when they are defined by crystal symmetry. An approximate (isotropic) treatment of cell esds is used for estimating esds involving l.s. planes.
Refinement. Refinement of F^2^ against ALL reflections. The weighted R-factor wR and goodness of fit S are based on F^2^, conventional R-factors R are based on F, with F set to zero for negative F^2^. The threshold expression of F^2^ > 2sigma(F^2^) is used only for calculating R-factors(gt) etc. and is not relevant to the choice of reflections for refinement. R-factors based on F^2^ are statistically about twice as large as those based on F, and R- factors based on ALL data will be even larger.

### Fractional atomic coordinates and isotropic or equivalent isotropic displacement parameters (Å^2^)

**Table:**

	*x*	*y*	*z*	*U*_iso_*/*U*_eq_	
Br	0.80657 (3)	1.10273 (9)	0.53574 (4)	0.03592 (18)	
S2	0.47258 (6)	0.4834 (2)	0.39045 (8)	0.0260 (3)	
O1	0.60773 (18)	0.2770 (7)	0.6641 (2)	0.0234 (7)	
C2	0.6253 (2)	0.5717 (8)	0.5481 (3)	0.0210 (9)	
C1	0.5474 (2)	0.4374 (8)	0.5090 (3)	0.0196 (8)	
C3	0.6679 (2)	0.7694 (9)	0.5131 (3)	0.0226 (8)	
H3	0.6470	0.8467	0.4497	0.027*	
C9	0.7699 (3)	0.4064 (11)	0.8116 (4)	0.0362 (13)	
H9A	0.7768	0.2220	0.8027	0.043*	
H9B	0.7326	0.4324	0.8514	0.043*	
H9C	0.8225	0.4832	0.8457	0.043*	
C8	0.5396 (2)	0.2678 (9)	0.5812 (3)	0.0222 (9)	
C6	0.7356 (3)	0.5336 (10)	0.7103 (3)	0.0253 (9)	
C4	0.7437 (3)	0.8416 (9)	0.5800 (4)	0.0261 (9)	
C7	0.6596 (2)	0.4648 (9)	0.6435 (3)	0.0215 (8)	
C5	0.7778 (3)	0.7289 (10)	0.6741 (4)	0.0283 (10)	
H5	0.8294	0.7837	0.7139	0.034*	
O2	0.4684 (2)	0.7724 (8)	0.3685 (3)	0.0389 (10)	
C10	0.4738 (3)	0.0806 (9)	0.5865 (4)	0.0279 (10)	
H10A	0.4286	0.0927	0.5255	0.042*	
H10B	0.4548	0.1227	0.6437	0.042*	
H10C	0.4955	−0.0946	0.5937	0.042*	
C11	0.5294 (4)	0.3399 (11)	0.3125 (4)	0.0361 (12)	
H11A	0.4998	0.3659	0.2425	0.054*	
H11B	0.5362	0.1555	0.3264	0.054*	
H11C	0.5827	0.4216	0.3271	0.054*	

### Atomic displacement parameters (Å^2^)

**Table:**

	*U*^11^	*U*^22^	*U*^33^	*U*^12^	*U*^13^	*U*^23^
Br	0.0297 (2)	0.0241 (2)	0.0578 (3)	−0.00886 (19)	0.01865 (19)	−0.0052 (3)
S2	0.0213 (5)	0.0276 (6)	0.0247 (5)	−0.0042 (4)	−0.0002 (4)	0.0032 (4)
O1	0.0239 (15)	0.0250 (17)	0.0218 (15)	−0.0003 (12)	0.0075 (12)	0.0008 (13)
C2	0.018 (2)	0.019 (2)	0.025 (2)	0.0008 (14)	0.0051 (16)	−0.0027 (16)
C1	0.0152 (17)	0.0208 (19)	0.0210 (19)	−0.0012 (14)	0.0024 (14)	0.0019 (16)
C3	0.0211 (19)	0.020 (2)	0.026 (2)	0.0005 (15)	0.0066 (16)	0.0004 (16)
C9	0.027 (2)	0.051 (4)	0.026 (3)	0.007 (2)	0.0003 (19)	−0.002 (2)
C8	0.0208 (18)	0.024 (2)	0.021 (2)	0.0003 (15)	0.0043 (16)	−0.0022 (16)
C6	0.0197 (19)	0.027 (2)	0.027 (2)	0.0039 (16)	0.0047 (16)	−0.0055 (19)
C4	0.0216 (19)	0.0183 (19)	0.040 (3)	−0.0028 (15)	0.0121 (17)	−0.0054 (19)
C7	0.0190 (18)	0.021 (2)	0.025 (2)	0.0022 (15)	0.0071 (15)	−0.0030 (18)
C5	0.0176 (18)	0.034 (3)	0.032 (2)	−0.0017 (16)	0.0051 (16)	−0.012 (2)
O2	0.041 (2)	0.0286 (19)	0.039 (2)	0.0081 (16)	−0.0001 (18)	0.0065 (16)
C10	0.027 (2)	0.023 (2)	0.035 (2)	−0.0066 (17)	0.0121 (19)	0.0006 (18)
C11	0.052 (3)	0.031 (3)	0.026 (2)	−0.007 (2)	0.012 (2)	−0.002 (2)

### Geometric parameters (Å, °)

**Table:**

Br—C4	1.913 (5)	C9—H9B	0.9600
S2—O2	1.502 (4)	C9—H9C	0.9600
S2—C1	1.769 (4)	C8—C10	1.486 (6)
S2—C11	1.795 (7)	C6—C7	1.391 (6)
O1—C8	1.368 (5)	C6—C5	1.400 (8)
O1—C7	1.384 (6)	C4—C5	1.383 (8)
C2—C7	1.387 (6)	C5—H5	0.9300
C2—C3	1.404 (7)	C10—H10A	0.9600
C2—C1	1.444 (5)	C10—H10B	0.9600
C1—C8	1.354 (7)	C10—H10C	0.9600
C3—C4	1.394 (6)	C11—H11A	0.9600
C3—H3	0.9300	C11—H11B	0.9600
C9—C6	1.496 (7)	C11—H11C	0.9600
C9—H9A	0.9600		
			
O2—S2—C1	107.2 (2)	C7—C6—C9	122.8 (5)
O2—S2—C11	106.4 (3)	C5—C6—C9	122.9 (4)
C1—S2—C11	97.8 (2)	C5—C4—C3	124.3 (5)
C8—O1—C7	106.4 (3)	C5—C4—Br	118.1 (3)
C7—C2—C3	119.6 (4)	C3—C4—Br	117.6 (4)
C7—C2—C1	104.6 (4)	O1—C7—C2	110.6 (3)
C3—C2—C1	135.8 (4)	O1—C7—C6	123.9 (4)
C8—C1—C2	107.6 (4)	C2—C7—C6	125.5 (5)
C8—C1—S2	124.6 (3)	C4—C5—C6	121.0 (4)
C2—C1—S2	127.7 (4)	C4—C5—H5	119.5
C4—C3—C2	115.3 (4)	C6—C5—H5	119.5
C4—C3—H3	122.4	C8—C10—H10A	109.5
C2—C3—H3	122.4	C8—C10—H10B	109.5
C6—C9—H9A	109.5	H10A—C10—H10B	109.5
C6—C9—H9B	109.5	C8—C10—H10C	109.5
H9A—C9—H9B	109.5	H10A—C10—H10C	109.5
C6—C9—H9C	109.5	H10B—C10—H10C	109.5
H9A—C9—H9C	109.5	S2—C11—H11A	109.5
H9B—C9—H9C	109.5	S2—C11—H11B	109.5
C1—C8—O1	110.8 (4)	H11A—C11—H11B	109.5
C1—C8—C10	133.0 (4)	S2—C11—H11C	109.5
O1—C8—C10	116.2 (4)	H11A—C11—H11C	109.5
C7—C6—C5	114.3 (4)	H11B—C11—H11C	109.5
			
C7—C2—C1—C8	0.8 (5)	C2—C3—C4—C5	1.1 (7)
C3—C2—C1—C8	−178.2 (5)	C2—C3—C4—Br	178.2 (3)
C7—C2—C1—S2	179.5 (3)	C8—O1—C7—C2	−0.7 (5)
C3—C2—C1—S2	0.6 (8)	C8—O1—C7—C6	179.5 (4)
O2—S2—C1—C8	139.5 (4)	C3—C2—C7—O1	179.1 (4)
C11—S2—C1—C8	−110.6 (5)	C1—C2—C7—O1	−0.1 (5)
O2—S2—C1—C2	−39.0 (5)	C3—C2—C7—C6	−1.0 (7)
C11—S2—C1—C2	70.8 (5)	C1—C2—C7—C6	179.8 (4)
C7—C2—C3—C4	0.3 (7)	C5—C6—C7—O1	−179.8 (4)
C1—C2—C3—C4	179.1 (5)	C9—C6—C7—O1	1.1 (7)
C2—C1—C8—O1	−1.3 (5)	C5—C6—C7—C2	0.4 (7)
S2—C1—C8—O1	179.9 (3)	C9—C6—C7—C2	−178.7 (5)
C2—C1—C8—C10	179.1 (5)	C3—C4—C5—C6	−1.9 (8)
S2—C1—C8—C10	0.3 (8)	Br—C4—C5—C6	−179.0 (4)
C7—O1—C8—C1	1.2 (5)	C7—C6—C5—C4	1.0 (7)
C7—O1—C8—C10	−179.0 (4)	C9—C6—C5—C4	−179.9 (5)

### Hydrogen-bond geometry (Å, °)

**Table:**

*D*—H···*A*	*D*—H	H···*A*	*D*···*A*	*D*—H···*A*
C11—H11B···O2^i^	0.96	2.42	3.242 (7)	143

Symmetry codes: (i) *x*, *y*−1, *z*.
